# Reference Standard for Digital Infrared Thermography of the Surface Temperature of the Lower Limbs

**DOI:** 10.3390/bioengineering10030283

**Published:** 2023-02-21

**Authors:** Ho Yeol Zhang, Seong Son, Byung Rhae Yoo, Tae-Mi Youk

**Affiliations:** 1 Department of Neurosurgery, National Health Insurance Service Ilsan Hospital, Yonsei University College of Medicine, Ilsan 10444, Republic of Korea; 2 Department of Neurosurgery, Gil Medical Center, Gachon University College of Medicine, Incheon 21565, Republic of Korea; 3 Research Institute, National Health Insurance Service Ilsan Hospital, Yonsei University College of Medicine, Ilsan 10444, Republic of Korea; 4 Department of Statistics, Korea University, Seoul 02841, Republic of Korea

**Keywords:** infrared rays, lower limb, reference standard, skin temperature, thermography

## Abstract

Digital infrared thermographic imaging (DITI) is a supplementary diagnostic technique to visualize the surface temperature of the human body. However, there is currently no reference standard for the lower limbs for accurate diagnosis. In this study, we performed DITI on the lower limbs of 905 healthy Korean volunteers (411 males and 494 females aged between 20 and 69 years) to obtain reference standard data. Thermography was conducted on the front, back, lateral sides, and sole area, and 188 regions of interest (ROIs) were analyzed. Additionally, subgroup analysis was conducted according to the proximity of ROIs, sex, and age groups. The mean temperatures of ROIs ranged from 24.60 ± 5.06 to 28.75 ± 5.76 °C and the absolute value of the temperature difference between both sides reached up to 1.06 ± 2.75 °C. According to subgroup analysis, the sole area had a significantly lower temperature than any other areas, men had higher temperatures than women, and the elderly had higher temperatures than the young adults except for the 20s age group (*p* < 0.001, respectively). This result could be used as a foundation for the establishment of a reference standard for DITI. Practical patient DITI can be accurately interpreted using these data, and it can serve as a basis for further scientific research.

## 1. Introduction

Digital infrared thermographic imaging (DITI) is a technique used to display the body’s surface temperature using thermography [1,2]. DITI has been used as a complementary diagnostic tool in various clinical fields [3,4,5,6,7,8,9]. In several disorders involving the lower limbs, such as lumbar radicular pain, chronic regional pain syndrome, vascular disease, and peripheral nerve entrapment, DITI allows visualization of the affected area as hypo-radiant (hypothermia) or hyper-radiant (hyperthermia) compared to the unaffected area [10,11,12,13,14,15]. 

In terms of interpreting DITI, the normal range of skin temperature and criteria for hypo-radiant and hyper-radiant are ambiguous. Empirically, the temperature difference between both sides of the lower limbs is significant when it is more than 0.1–0.3 °C, depending on the location of the surface area [1,16,17]. However, a formally approved consensus on the definition of significant difference between both sides, as well as hypo-radiant or hyper-radiant, is still undetermined due to variations in equipment, environment (room temperature and humidity) of the test room, and the ability of the surveyor [5]. Furthermore, despite the consistent examination conditions, the range of normal surface temperature still varies according to baseline characteristics such as sex, age, body mass index, patient condition, and medical history [17]. 

To overcome these barriers to the definition of abnormality and to establish a correct standard for DITI, a standardized measurement protocol and a reference standard for DITI are necessary. However, there are no studies about reference standard data for DITI of the lower limbs. In this study, we performed DITI on a large group of healthy volunteers using a standardized protocol and provide detailed reference standard data for DITI of the lower extremities.

## 2. Materials and Methods

### 2.1. Trial Design and Ethics 

This multi-center, single-arm, open-label trial was conducted for 2 years in accordance with the 1964 Helsinki Declaration and its later amendments. All processes of the study were approved by the Institutional Review Board of three different research centers. Additionally, this research was registered as a clinical trial in the Clinical Research Information Service of the Republic of Korea (number KCT0006880).

### 2.2. Sample Size

The number of samples was calculated as follows: 


$$n\;=\;\frac{\theta {\left(1\;–\;\theta \right)}^{2}z_{\alpha /2}^{2}\;}{d^{2}}$$

The population proportion (*θ*) for the exam was set to 0.85 and the margin of error (*d*) was set to 0.025. Using the above formula with a significance level of 5% and a confidence level of 95%, a sample size of 784 was calculated. Considering a dropout rate of 15%, a total of 922 participants were necessary. 

### 2.3. Subjects

Healthy test subjects were voluntarily recruited through public announcements and tests were conducted at three institutions from March 2018 to December 2020.

To minimize bias related to subject selection and external effect, inclusion criteria were as follows: (1) adults between the ages of 20 and 69; (2) no specific medical history including diabetes mellitus, peripheral neuropathy, spinal stenosis, disc herniation, joint disease of the leg, previous surgery history of the spine and lower limbs, or recent trauma; (3) no definite present pain or skin lesion in the lower limbs; (4) no potential risk of test such as claustrophobia, pregnant, or lactating women; (5) who can maintain a stationary posture for the required amount of time during the test; (6) without any other reason for disqualification according to the judgment of the researchers. Participation was granted when the requirements were met based on the inclusion criteria questionnaire. 

A total of 922 healthy Korean volunteers were registered with an even distribution based on sex and age group. DITI was conducted following a standardized protocol and informed consent was obtained in advance from all participants. Among them, 905 participants were evaluated after excluding 17 participants due to measurement failure and/or withdrawal of consent.

### 2.4. Equipment and Examination Protocol

All examinations were conducted in outpatient clinics in three different hospitals. DITI was performed using the Iris-XP Digital infrared imaging system (Medicore, Seoul, Republic of Korea). Volunteers scheduled for DITI were informed about general precautions such as avoiding exposure to cold or hot environments, not smoking, and not consuming caffeine for 1 h before the test [18]. 

The skin temperature of subjects can be affected by environmental temperature and humidity due to sweating evaporation and vasoconstriction/vasodilation response [19,20,21]. To maintain consistency, we controlled the air temperature and humidity in the test room. Specifically, the room temperature and humidity were maintained at 20.0–23.0 °C and 30–75%, respectively. After undressing completely, the subjects remained in the room for approximately 20 min to acclimate prior to the examination. They were allowed to stand or sit on a chair with a back, depending on their preference.

The measurement reliability of temperature using the DITI equipment was found to be reasonable. The uncertainty of the thermography equipment ranged from 0.000 °C to 0.369 °C, as specified by the Korean Agency for Technology and Standards. 

**Figure 1:**
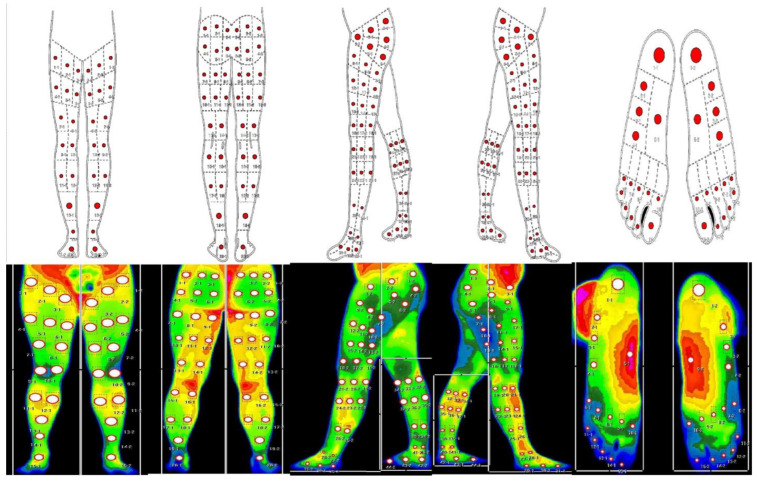
Diagram and practical imaging of regions of interest in the front, lateral-side, back, and sole areas of the leg.

The test was conducted in the front area, back area including buttocks, both lateral-side areas, and the sole area of both feet. A total of 188 regions of interest (ROIs) were manually divided into 15 × 2 ROIs in the front area, 44 × 2 ROIs in both lateral-side areas, 20 × 2 ROIs in the back area, and 15 × 2 ROIs in the sole area (Figure 1). To ensure the accuracy of ROI division and measurement, objective testing and diagraming of pictures based on 188 ROIs of all subjects were performed by five certified surveyors. 

### 2.5. Statistical Analysis

A quantitative analysis of data was conducted by a specialized doctor and a statistician who was blinded to participant information. 

The analysis was performed using SPSS version 27.0 (IBM Corporation, Armonk, NY, USA). The normal distribution of the data was evaluated using the Kolmogorov–Smirnov test, and all data were reported as mean ± standard deviation or mean with 95% confidence intervals (CI). One-way analysis of variance (ANOVA), linear regression analysis, and paired t-tests were performed according to the characteristics of the values. Statistical significance was accepted at *p* < 0.05.

## 3. Results

### 3.1. Subjects

The mean age of all participants (n = 905) was 42.86 ± 12.87 years, and 45.4% of the participants were male (n = 411). The demographic distribution of volunteers according to age group was as follows: 183 (97 males and 86 females) in their 20s; 213 (108 males and 105 females) in their 30s; 228 (109 males and 119 females) in their 40s; 177 (65 males and 112 females) in their 50s; 104 (32 males and 72 females) in their 60s.

### 3.2. Overall Data: The Mean Temperature and Difference between Both Sides (°C)

The mean temperature of the ROIs of each area and the temperature difference between both sides (ΔT, right—left) were as follows: in the front area, the overall mean temperature was 27.69 ± 5.34 (ranged from 26.73 ± 5.12 (extended uncertainty 10.27) to 28.75 ± 5.76 (extended uncertainty 11.54)) and the overall mean difference was 0.03 ± 0.41 (ranged from −0.09 ± 0.33 to 0.24 ± 0.47); in the back area, the overall mean temperature was 27.70 ± 5.38 (ranged from 25.74 ± 5.09 (extended uncertainty 10.21) to 28.48 ± 5.74 (extended uncertainty 11.50)) and the overall mean difference was −0.04 ± 0.42 (ranged from −0.23 ± 0.59 to 0.20 ± 0.42); in the lateral-side area, the overall mean temperature was 27.18 ± 5.49 (ranged from 25.56 ± 5.19 (extended uncertainty 10.41) to 28.53 ± 5.79 (extended uncertainty 11.60)) and the overall mean difference was −0.58 ± 2.72 (ranged from −1.06 ± 2.75 to −0.09 ± 2.37); in the sole area, the overall mean temperature was 25.74 ± 4.98 (ranged from 24.60 ± 5.06 (extended uncertainty 10.1) to 27.69 ± 5.31 (extended uncertainty 10.65)) and the overall mean difference was −0.09 ± 0.80 (ranged from −0.17 ± 0.74 to 0.06 ± 0.71) (Table 1, Table 2, Table 3 and Table 4).

**Figure 2:**
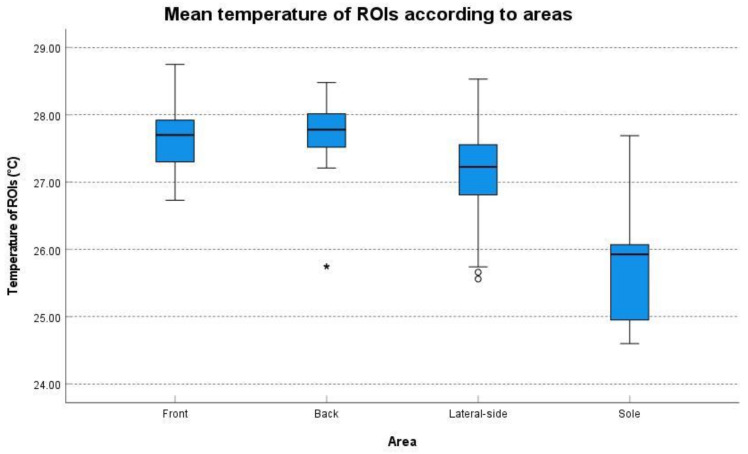
Diagram of the mean temperature according to the four areas. ROIs: region of interests; and * and ⸰ are labels that are out of range.

According to the location of the ROIs, the mean temperature of the ROIs was significantly different between the four areas (*p* < 0.001, ANOVA). In particular, the temperature of the sole area was significantly lower than that of any other areas; the difference between the sole and other areas was 1.93 (95% CI, 1.53–2.33), 1.95 (95% CI, 1.57–2.32), and 1.41 (95% CI, 1.08–1.74), respectively (*p* < 0.001, ANOVA post hoc analysis) (Figure 2). 

**Figure 3:**
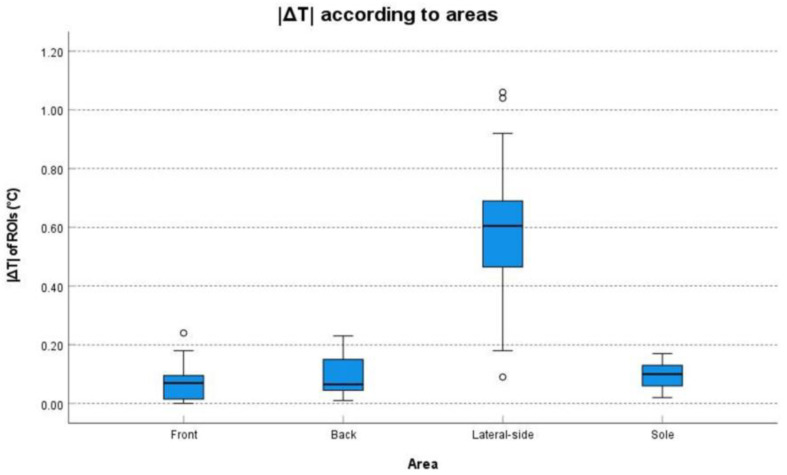
Diagram of the absolute values of difference between both sides (|ΔT|, |right—left|) in each of the four areas. ROIs: region of interests; and ⸰ are labels that are out of range.

Moreover, the absolute value of the temperature difference between both sides (|ΔT|) was also significantly different between the four areas (*p* < 0.001, ANOVA). In particular, the |ΔT| of the lateral-side area was 0.58 ± 2.72 °C (95% CI, 0.40–0.76), which was significantly larger compared to any other area; the mean difference of |ΔT| between lateral-side and other areas was 0.50 (95% CI, 0.38–0.63), 0.48 (95% CI, 0.37–0.60), and 0.48 (95% CI, 0.36–0.60), respectively (*p* < 0.001, ANOVA post hoc analysis) (Figure 3).

### 3.3. Subgroup Analysis of the Temperature (°C) According to Proximity of ROIs 

**Figure 4:**
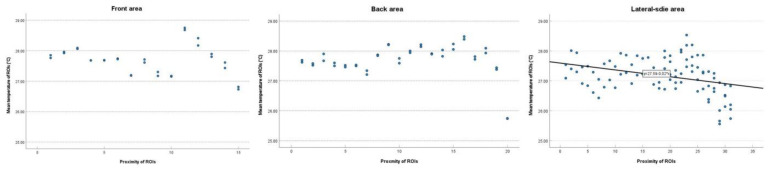
Relation between the mean temperature of the region of interest (ROIs) and the proximity of ROIs in three areas according to regression analysis. Front area temperature = 27.871 − (0.023 × ROIs) (*p* = 0.260, R^2^ = 0.045); back area temperature = 27.792 − (0.009 × ROIs) (*p* = 0.569, R^2^ = 0.009); lateral-side area temperature = 27.595 − (0.023 × ROIs) (*p* = 0.001, R^2^ = 0.114).

The temperature tended to drop from the proximal to the distal part in only the lateral-side area (*p* = 0.001, regression analysis), not in the front or back areas (Figure 4).

**Figure 5:**
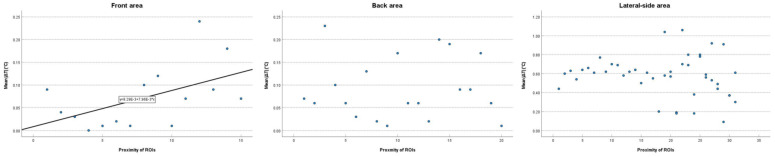
Relation between the absolute values of difference between both sides (|ΔT|, |right—left|) and the proximity of region of interest in each of the three areas according to regression analysis. |ΔT| front area = 0.008 + (0.008 × ROIs) (*p* = 0.047, R^2^ = 0.270); |ΔT| back area = 0.090 + (9.774 × 10^−5^ × ROIs) (*p* = 0.972, R^2^ = 0.000); |ΔT| lateral-side area = 0.645 − (0.004 × ROIs) (*p* = 0.327, R^2^ = 0.023).

In terms of |ΔT| according to the proximity of ROIs, the |ΔT| tended to increase from the proximal to the distal part in only the front area (*p* = 0.047, regression analysis), not in the back or lateral-side areas (Figure 5). 

### 3.4. Subgroup Analysis of the Temperature (°C) According to Sex

The mean temperature of each area depending on the sex was as follows: in the front area, the mean temperature was 28.83 ± 5.15 (ranged from 27.10 ± 5.16 to 32.02 ± 4.85) in males and 26.54 ± 5.53 (ranged from 24.03 ± 5.16 to 29.32 ± 5.66) in females; in the back area, the mean temperature was 28.90 ± 5.18 (ranged from 25.99 ± 5.05 to 31.84 ± 4.65) in males and 26.50 ± 5.61 (ranged from 22.86 ± 4.86 to 28.74 ± 5.59) in females; in the lateral-side area, the mean temperature was 27.99 ± 5.45 (ranged from 25.32 ± 5.25 to 32.15 ± 4.71 in males and 26.29 ± 5.55 (ranged from 22.89 ± 4.95 to 29.02 ± 5.58) in females; in the sole area, the mean temperature was 26.59 ± 4.87 (ranged from 25.32 ± 5.08 to 30.36 ± 5.03) in males and 24.85 ± 5.03 (ranged from 22.19 ± 4.69 to 28.21 ± 5.40) in females; and in all areas, the mean temperature was 28.08 ± 5.16 in males and 26.05 ± 5.43 in females (Table 1). 

**Figure 6:**
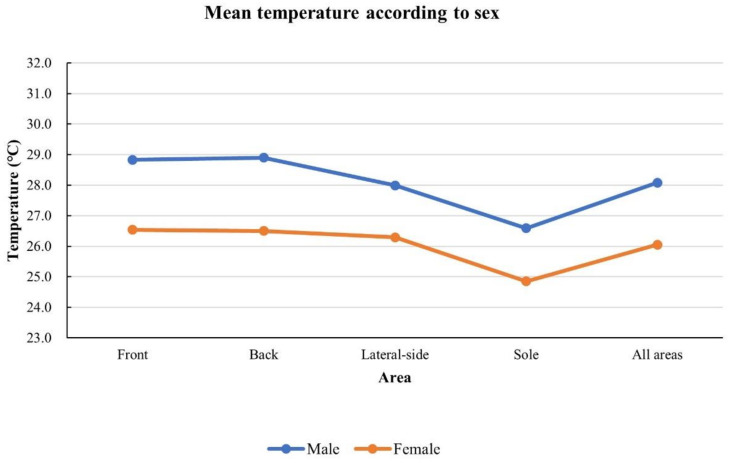
Trend of temperature according to sex in each area.

In terms of trends according to sex, the mean surface temperature in the same ROIs was higher in males than in females in all areas of all age groups (*p* < 0.001, paired t-test), although the mean differences varied depending on the areas and age groups. The males’ surface temperatures were at least 0.52 (95% CI, 0.09–0.94) and at most 3.42 (95% CI, 3.19–3.65) higher than that of females (Table 5 and Figure 6).

### 3.5. Subgroup Analysis of the Temperature (°C) According to Age Group

The mean temperature of each area depending on the age group was as follows: in the 20s age group, the mean temperature was 27.34 ± 5.17 (ranged from 24.14 ± 4.69 to 29.33 ± 5.21); in the 30s age group, the mean temperature was 25.82 ± 5.37 (ranged from 22.19 ± 4.69 to 28.95 ± 5.74); in the 40s age group, the mean temperature was 26.21 ± 5.71 (ranged from 23.74 ± 5.30 to 30.00 ± 5.43); in the 50s age group, the mean temperature was 27.65 ± 5.11 (ranged from 24.67 ± 5.16 to 30.84 ± 4.95); in the 60s age group, the mean temperature was 28.20 ± 5.29 (ranged from 25.06 ± 5.34 to 32.15 ± 4.85) (Table 5).

**Figure 7:**
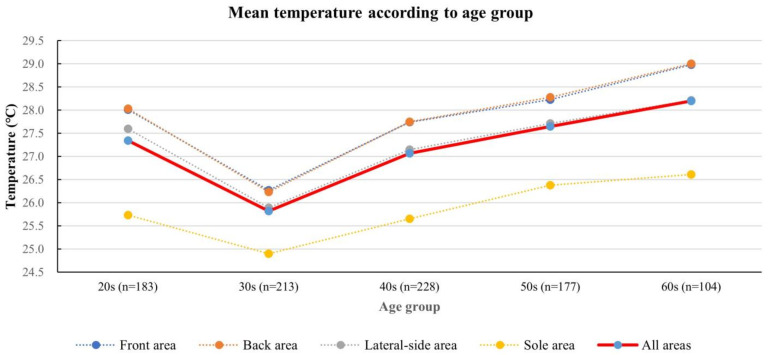
Trend of temperature according to age group in each area.

In terms of trends according to age group, the surface temperature increased as age increased, except for the 20s age group, in all areas (*p* < 0.001, ANOVA). Among all age groups, the 30s age group of both sexes showed the lowest temperature in all areas (*p*< 0.001, ANOVA post hoc analysis) (Table 5 and Figure 7). 

## 4. Discussion

### 4.1. The Mean Temperature and Difference between Both Sides

The surface temperature of the lower limb was difficult to define as a single numerical average because the mean temperature of ROIs was significantly different between the areas and location of ROIs (ranging from 24.60 ± 5.06 °C to 28.75 ± 5.76 °C). Notably, the temperature of the sole area was significantly lower than that of other areas. This finding supports the previous suggestion that distal skin regions, including feet and hands, are hypo-radiant areas because they are further away from the body’s main thermal cores, such as the great vessels and viscera [22,23]. 

In previous clinical studies, the normal range of |ΔT| was limited to 0.2 °C, although it varied depending on the region [10,15,17]. According to the present study, |ΔT| was within 0.2 °C, as suggested previously in almost all ROIs except in the lateral-side area. In the lateral-side area, |ΔT| was higher than 0.2 °C at 0.58 ± 2.72 °C (95% CI, 0.40–0.76). The reason for this variability requires further study, and careful interpretation should be taken when evaluating |ΔT| in the lateral-side area.

The results of this study, which include mean temperature and mean ΔT of each ROI from a large sample, can serve as a reference standard in DITI. Based on this reference standard, objective hypo-radiant/hyper-radiant and clinical significance of ΔT can be determined by comparing the practical DITI results with this reference data. However, it may be difficult to subdivide the ROIs in practical clinics as was done in this study. In such cases, abbreviations for representative RIOs can be used and analyzed. It is important to compare values in each ROI of each area, rather than relying on simple overall averages. Additionally, because various individual characteristics, such as sex, age, medical condition, body composition, and circadian rhythm, can affect the skin temperature [24,25,26,27], normal values for an individual may fall outside the range of reference standards from this study. Therefore, further research is needed to assess the sensitivity and specificity of this reference standard by applying it to actual patients in the future. 

### 4.2. Subgroup Analysis

We investigated subgroup analysis, including the trend of surface temperature according to the proximity of ROIs, sex, and age groups, to verify existing claims and controversies related to surface temperature. A previous suggestion that the surface temperature decreases and |ΔT| increases from the proximal to the distal part was limited to only certain areas. Additionally, it was confirmed that men have significantly higher surface temperatures than women in all areas and in all age groups. In terms of the trend of surface temperature related to age, a complex phenomenon that the temperature increased with age except for the 20s age group concluded several existing controversies. 

This study’s findings that the surface temperature in the lateral-side area decreases from the proximal to the distal part and that the temperature of the sole is the lowest among the four areas are consistent with existing hypotheses. Previous studies suggested that the body temperature would drop from the proximal part to the distal part of the body [6]. We speculate that the reason for this phenomenon is that the bloodstream temperature of the proximal part is warmer than that of the distal part. However, in the front and back areas, this hypothesis does not apply and is, therefore, still controversial.

Based on the results of this study, |ΔT| increased toward the distal part in the front area only, but not in other areas. Furthermore, |ΔT| was within 0.2 °C in the sole area and it was measured as higher than 0.2 in the lateral-side area. In contrast, some authors have suggested that |ΔT| increases toward the distal part of the body and that the normal range of |ΔT| can be larger than 0.2 °C in the periphery [10,17]. As a result, previous findings are controversial, and it is necessary to be careful about the interpretation of the significance of |ΔT| depending on the area. 

According to this study, there was a significant trend that the surface temperature of females was lower than that of males in all areas of lower limbs. The influence of sex on surface temperature has been a subject of controversy. Some studies have reported that sex differences were significant for only certain ROIs, while others have suggested that women have lower skin temperatures [24]. This study concludes the previous controversy in this regard. A possible explanation is that the higher level of subcutaneous adipose tissue in women is associated with decreased surface temperature [28]. Greater subcutaneous fat in females may provide a significant insulating layer that blunts heat transfer from the core of the body or major blood vessels [29].

In terms of the relationship between surface temperature and aging, there has been controversy. Several studies have identified lower surface temperatures in the elderly and suggested that elderly individuals may have a lower skin temperature due to the association of aging with the loss of muscle mass, which reduces metabolism and limits heat generation [30,31,32]. In contrast, other studies have found that the elderly tend to have similar or higher skin temperature than young people and insisted that the blood flow of the human skin is controlled by the sympathetic nervous system innervation, which is mainly affected by the aging process [33,34,35]. 

According to the present study, the trend of surface temperature across age groups revealed a complicated pattern. Specifically, although the surface temperature tended to increase with age, the surface temperature in the 20s age group was paradoxically higher than that in the 30s age group. Consequently, individuals in their 30s showed the lowest temperature compared to all other age groups in both sexes. This trend may suggest that body surface temperature can vary according to age, even within the same individual. 

Aging is related to the alteration of the cutaneous vasodilation and vasoconstriction reflexes that modify peripheral circulation [3]. In other words, older adults have a higher skin temperature due to increased blood flow in the skin caused by a deficit in the venous return [36,37]. On the other hand, the higher metabolic rate of the muscle tissue and active reproductive organs with higher blood supply could have a positive influence on the higher surface temperature of young adults, especially the 20s age group [30,31]. We presume that the 30s age group has a paradoxical heat loss (heat redistribution) in the subcutaneous or skin layer, although they also have a high core temperature. Abundant subcutaneous fat in the 30s age group compared to the 20s age group could lower skin temperature by blocking heat transfer from the core of the body or major blood vessels [29]. In summary, we assume that the trend of body surface temperature according to age reveals a complicated pattern due to complex physiological phenomena related to heat generation and heat redistribution according to the aging process.

### 4.3. Limitations

This study has some limitations regarding various intrinsic or extrinsic factors that can influence surface temperature [38,39]. Firstly, the environment of the subjects was not entirely controlled during the DITI exam. All physiological and psychological factors, such as time of the test, circadian rhythm, menstrual cycle, menopause, emotional stress, and season, that can affect autonomic function, were neither controlled nor collected. Secondly, the control range of the test room temperature and humidity was too wide. We were unable to strictly control these factors due to practical limitations in the test room environment and seasonal variations. Thirdly, other demographic factors, such as ethnic variability, body mass index, and body fat percentage, were not considered.

However, we tried to minimize errors by limiting the inclusion criteria to healthy subjects with no specific medical history or symptoms, although healthy volunteers did not rule out the possibility of varicose veins, knee osteoarthritis, scars on the lower limbs, limb shortening, postural deviations, or extra hair on the legs. Additionally, a large number of participants who underwent consistent measurement techniques could strengthen the reliability of our examination and analysis. To the best of our knowledge, this study is the first report to investigate the reference standard data of DITI.

## 5. Conclusions

This large-scale study of DITI of the lower limb could serve as a basis for establishing a reference standard for DITI. Additionally, we were able to analyze the trend of the surface temperature of lower limbs in terms of the proximity of ROIs, sex, and age. Using these reference standards and trends, DITI results in various diseases can be assessed through comparative analysis. Furthermore, the results of the present study can be used in the development of diagnosis using deep learning-based artificial intelligence in the future.

## Figures and Tables

**Table 1:** Overall mean temperature of regions of interest in the front area.

ROI	Mean (°C)	SD	Extended Uncertainty	Difference (ΔT, Right Side—Left Side)				
				Mean (°C)	SD	95% CI Lower	95% CI Upper	Extended Uncertainty
1_1	27.76	5.63	11.28					
1_2	27.85	5.65	11.32	−0.09	0.33	−0.11	−0.07	0.99
2_1	27.92	5.65	11.32					
2_2	27.96	5.67	11.36	−0.04	0.28	−0.06	−0.02	0.93
3_1	28.06	5.67	11.36					
3_2	28.09	5.75	11.52	−0.03	0.43	−0.05	0.00	1.13
4_1	27.68	5.58	11.18					
4_2	27.68	5.54	11.10	0.00	0.30	−0.02	0.02	0.95
5_1	27.68	5.57	11.16					
5_2	27.69	5.58	11.18	−0.01	0.29	−0.03	0.01	0.94
6_1	27.74	5.57	11.16					
6_2	27.72	5.58	11.18	0.02	0.33	0.00	0.05	0.99
7_1	27.19	5.42	10.87					
7_2	27.18	5.40	10.83	0.01	0.36	−0.01	0.03	1.03
8_1	27.61	5.49	11.00					
8_2	27.71	5.58	11.18	−0.10	0.36	−0.12	−0.07	1.03
9_1	27.30	5.33	10.69					
9_2	27.17	5.26	10.55	0.12	0.58	0.09	0.16	1.37
10_1	27.15	5.34	10.71					
10_2	27.17	5.40	10.83	−0.01	0.53	−0.05	0.02	1.29
11_1	28.68	5.71	11.44					
11_2	28.75	5.76	11.54	−0.07	0.38	−0.09	−0.04	1.06
12_1	28.41	5.73	11.48					
12_2	28.17	5.65	11.32	0.24	0.47	0.21	0.27	1.20
13_1	27.89	5.55	11.12					
13_2	27.80	5.53	11.08	0.09	0.40	0.07	0.12	1.09
14_1	27.61	5.48	10.98					
14_2	27.43	5.40	10.83	0.18	0.51	0.15	0.21	1.26
15_1	26.81	5.19	10.41					
15_2	26.73	5.12	10.27	0.07	0.65	0.03	0.11	1.49
Mean	27.69	5.34		0.03	0.41	0.00	0.06	

CI: confidence interval; ROI: region of interest; SD: standard deviation.

**Table 2:** Overall mean temperature of regions of interest in the back area.

ROI	Mean (°C)	SD	Extended Uncertainty	Difference (ΔT, Right Side—Left Side)				
				Mean (°C)	SD	95% CI Lower	95% CI Upper	Extended Uncertainty
1_1	27.62	5.69	11.40					
1_2	27.69	5.69	11.40	−0.07	0.53	−0.11	−0.04	1.29
2_1	27.52	5.67	11.36					
2_2	27.58	5.67	11.36	−0.06	0.40	−0.09	−0.03	1.09
3_1	27.67	5.64	11.30					
3_2	27.90	5.76	11.54	−0.23	0.59	−0.27	−0.19	1.39
4_1	27.50	5.61	11.24					
4_2	27.60	5.62	11.26	−0.10	0.49	−0.13	−0.06	1.23
5_1	27.52	5.59	11.20					
5_2	27.46	5.56	11.14	0.06	0.36	0.03	0.08	1.03
6_1	27.50	5.57	11.16					
6_2	27.53	5.59	11.20	−0.03	0.39	−0.06	−0.01	1.07
7_1	27.21	5.49	11.00					
7_2	27.34	5.52	11.06	−0.13	0.46	−0.16	−0.10	1.18
8_1	27.87	5.61	11.24					
8_2	27.84	5.60	11.22	0.02	0.36	0.00	0.05	1.03
9_1	28.22	5.66	11.34					
9_2	28.20	5.68	11.38	0.01	0.36	−0.01	0.04	1.03
10_1	27.59	5.54	11.10					
10_2	27.75	5.58	11.18	−0.17	0.41	−0.19	−0.14	1.10
11_1	27.94	5.61	11.24					
11_2	28.00	5.61	11.24	−0.06	0.33	−0.08	−0.03	0.99
12_1	28.14	5.64	11.30					
12_2	28.21	5.67	11.36	−0.06	0.35	−0.09	−0.04	1.02
13_1	27.91	5.61	11.24					
13_2	27.89	5.59	11.20	0.02	0.32	0.00	0.04	0.98
14_1	28.03	5.67	11.36					
14_2	27.82	5.60	11.22	0.20	0.42	0.18	0.23	1.12
15_1	28.05	5.63	11.28					
15_2	28.23	5.68	11.38	−0.19	0.32	−0.21	−0.17	0.98
16_1	28.48	5.74	11.50					
16_2	28.39	5.72	11.46	0.09	0.42	0.07	0.12	1.12
17_1	27.81	5.60	11.22					
17_2	27.72	5.56	11.14	0.09	0.38	0.06	0.11	1.06
18_1	27.93	5.59	11.20					
18_2	28.09	5.63	11.28	−0.17	0.43	−0.20	−0.14	1.13
19_1	27.38	5.50	11.02					
19_2	27.44	5.51	11.04	−0.06	0.42	−0.09	−0.03	1.12
20_1	25.74	5.09	10.21					
20_2	25.75	5.06	10.15	−0.01	0.65	−0.05	0.03	1.49
Mean	27.70	5.38		−0.04	0.42	−0.07	−0.01	

CI: confidence interval; ROI: region of interest; SD: standard deviation.

**Table 3:** Overall mean temperature of regions of interest in the lateral-side area.

ROI	Mean (°C)	SD	Extended Uncertainty	Difference (ΔT, Right Side—Left Side)				
				Mean (°C)	SD	95% CI Lower	95% CI Upper	Extended Uncertainty
1_1	27.09	5.90	11.82					
1_2	27.54	5.73	11.48	−0.44	2.84	−0.63	−0.26	5.73
2_1	27.40	5.97	11.96					
2_2	28.01	5.85	11.72	−0.60	2.93	−0.79	−0.41	5.91
3_1	27.30	5.92	11.86					
3_2	27.94	5.78	11.58	−0.63	2.95	−0.82	−0.43	5.95
4_1	26.91	5.81	11.64					
4_2	27.46	5.69	11.40	−0.54	2.83	−0.72	−0.35	5.71
5_1	26.84	5.81	11.64					
5_2	27.49	5.69	11.40	−0.64	2.91	−0.83	−0.45	5.87
6_1	26.61	5.65	11.32					
6_2	27.29	5.58	11.18	−0.66	2.75	−0.84	−0.48	5.55
7_1	26.43	5.60	11.22					
7_2	27.05	5.54	11.10	−0.61	2.74	−0.78	−0.43	5.53
8_1	26.79	5.73	11.48					
8_2	27.57	5.70	11.42	−0.77	2.85	−0.95	−0.58	5.75
9_1	27.03	5.81	11.64					
9_2	27.67	5.70	11.42	−0.62	2.80	−0.81	−0.44	5.65
10_1	26.77	5.70	11.42					
10_2	27.48	5.68	11.38	−0.70	2.81	−0.89	−0.52	5.67
11_1	27.22	5.81	11.64					
11_2	27.92	5.74	11.50	−0.69	2.84	−0.88	−0.51	5.73
12_1	27.27	5.86	11.74					
12_2	27.86	5.70	11.42	−0.58	2.85	−0.76	−0.39	5.75
13_1	26.91	5.71	11.44					
13_2	27.54	5.61	11.24	−0.62	2.75	−0.80	−0.44	5.55
14_1	27.19	5.79	11.60					
14_2	27.84	5.68	11.38	−0.64	2.83	−0.83	−0.46	5.71
15_1	27.23	5.78	11.58					
15_2	27.75	5.63	11.28	−0.50	2.83	−0.69	−0.32	5.71
16_1	27.16	5.75	11.52					
16_2	27.78	5.61	11.24	−0.61	2.77	−0.79	−0.42	5.59
17_1	26.88	5.68	11.38					
17_2	27.44	5.56	11.14	−0.55	2.76	−0.73	−0.37	5.57
18_1	26.75	5.63	11.28					
18_2	26.95	5.40	10.83	−0.20	2.71	−0.37	−0.02	5.47
19_1	27.41	5.86	11.74					
19_2	28.00	5.69	11.40	−0.58	2.75	−0.76	−0.40	5.55
20_1	27.21	5.73	11.48					
20_2	27.84	5.60	11.22	−0.62	2.64	−0.79	−0.44	5.33
21_1	27.14	5.71	11.44					
21_2	27.35	5.43	10.89	−0.19	2.73	−0.37	−0.01	5.51
22_1	27.24	5.77	11.56					
22_2	27.96	5.69	11.40	−0.70	2.73	−0.88	−0.52	5.51
23_1	27.71	5.89	11.80					
23_2	28.53	5.79	11.60	−0.80	2.86	−0.99	−0.62	5.77
24_1	27.80	5.92	11.86					
24_2	28.20	5.68	11.38	−0.38	2.91	−0.57	−0.19	5.87
25_1	27.04	5.75	11.52					
25_2	27.86	5.69	11.40	−0.80	2.79	−0.98	−0.62	5.63
26_1	27.26	5.81	11.64					
26_2	27.86	5.67	11.36	−0.59	2.79	−0.77	−0.41	5.63
27_1	26.29	5.45	10.92					
27_2	26.83	5.42	10.87	−0.53	2.44	−0.69	−0.37	4.94
28_1	26.75	5.52	11.06					
28_2	27.25	5.38	10.79	−0.49	2.51	−0.65	−0.33	5.07
29_1	25.56	5.19	10.41					
29_2	25.66	5.09	10.21	−0.09	2.37	−0.24	0.07	4.80
30_1	26.14	5.34	10.71					
30_2	26.52	5.30	10.63	−0.37	2.42	−0.52	−0.21	4.90
31_1	26.20	5.35	10.73					
31_2	26.83	5.35	10.73	−0.61	2.44	−0.77	−0.45	4.94
32_1	26.72	5.59	11.20					
32_2	27.78	5.66	11.34	−1.04	2.79	−1.22	−0.86	5.63
33_1	27.04	5.67	11.36					
33_2	27.64	5.60	11.22	−0.57	2.81	−0.76	−0.39	5.67
34_1	26.74	5.59	11.20					
34_2	26.94	5.40	10.83	−0.18	2.80	−0.37	0.00	5.65
35_1	26.95	5.66	11.34					
35_2	28.04	5.71	11.44	−1.06	2.75	−1.24	−0.88	5.55
36_1	27.47	5.83	11.68					
36_2	28.19	5.72	11.46	−0.69	2.82	−0.88	−0.51	5.69
37_1	27.31	5.78	11.58					
37_2	27.51	5.49	11.00	−0.18	2.89	−0.37	0.01	5.83
38_1	26.65	5.62	11.26					
38_2	27.45	5.59	11.20	−0.78	2.68	−0.96	−0.61	5.41
39_1	26.73	5.64	11.30					
39_2	27.30	5.52	11.06	−0.56	2.70	−0.73	−0.38	5.45
40_1	26.38	5.34	10.71					
40_2	27.31	5.45	10.92	−0.92	2.41	−1.08	−0.76	4.88
41_1	26.64	5.50	11.02					
41_2	27.09	5.40	10.83	−0.44	2.61	−0.61	−0.27	5.27
42_1	26.01	5.31	10.65					
42_2	26.94	5.41	10.85	−0.91	2.44	−1.07	−0.75	4.94
43_1	26.49	5.41	10.85					
43_2	26.87	5.31	10.65	−0.37	2.47	−0.53	−0.21	4.99
44_1	25.74	5.25	10.53					
44_2	26.05	5.13	10.29	−0.30	2.41	−0.46	−0.14	4.88
Mean	27.18	5.49		−0.58	2.72	−0.76	−0.40	

CI: confidence interval; ROI: region of interest; SD: standard deviation.

**Table 4:** Overall mean temperature of regions of interest in the sole area.

ROI	Mean (°C)	SD	Extended Uncertainty	Difference (ΔT, Right Side—Left Side)				
				Mean (°C)	SD	95% CI Lower	95% CI Upper	Extended Uncertainty
1_1	26.05	5.12	10.27					
1_2	26.07	5.12	10.27	−0.02	0.86	−0.08	0.04	1.87
2_1	26.26	4.97	9.97					
2_2	26.36	5.02	10.07	−0.10	0.75	−0.15	−0.05	1.67
3_1	26.33	5.00	10.03					
3_2	26.42	5.03	10.09	−0.09	0.73	−0.14	−0.04	1.64
4_1	25.96	4.96	9.95					
4_2	26.13	5.00	10.03	−0.17	0.74	−0.22	−0.12	1.65
5_1	27.65	5.29	10.61					
5_2	27.69	5.31	10.65	−0.04	0.61	−0.08	0.00	1.43
6_1	25.69	5.07	10.17					
6_2	25.85	5.07	10.17	−0.15	0.87	−0.21	−0.10	1.89
7_1	25.87	5.12	10.27					
7_2	25.99	5.08	10.19	−0.12	0.76	−0.17	−0.07	1.69
8_1	25.93	5.07	10.17					
8_2	26.02	5.05	10.13	−0.09	0.72	−0.14	−0.04	1.62
9_1	25.99	5.03	10.09					
9_2	26.05	5.04	10.11	−0.06	0.71	−0.11	−0.01	1.60
10_1	25.92	5.04	10.11					
10_2	25.86	5.06	10.15	0.06	0.71	0.02	0.11	1.60
11_1	24.82	5.07	10.17					
11_2	24.95	5.07	10.17	−0.13	0.94	−0.20	−0.07	2.02
12_1	24.60	5.06	10.15					
12_2	24.77	5.06	10.15	−0.16	0.87	−0.22	−0.11	1.89
13_1	24.66	5.13	10.29					
13_2	24.79	5.11	10.25	−0.13	0.88	−0.19	−0.07	1.91
14_1	24.74	5.16	10.35					
14_2	24.84	5.16	10.35	−0.10	0.88	−0.16	−0.04	1.91
15_1	25.19	5.22	10.47					
15_2	25.24	5.24	10.51	−0.05	0.91	−0.11	0.01	1.96
Mean	25.74	4.98		−0.09	0.80	−0.14	−0.04	

CI: confidence interval; ROI: region of interest; SD: standard deviation.

**Table 5:** Comparison of mean temperature for the same region of interest according to sex and age group.

Characteristics	Male (°C)	Female (°C)	Mean (°C)	Difference Between Sexes (°C)	*p* Value
Front area					<0.001 ^a^
20s (n = 183)	28.46 ± 5.02	27.50 ± 5.68	28.01 ± 5.33	0.96 (95% CI, 0.70–1.21)	<0.001 ^b^
30s (n =213)	27.96 ± 5.51	24.54 ± 5.34	26.27 ± 5.43	3.42 (95% CI, 3.19–3.65)	<0.001 ^b^
40s (n = 228)	28.83 ± 5.18	26.74 ± 5.84	27.74 ± 5.52	2.09 (95% CI, 1.80–2.38)	<0.001 ^b^
50s (n = 177)	29.82 ± 4.91	27.31 ± 5.16	28.23 ± 5.07	2.51 (95% CI, 2.24–2.78)	<0.001 ^b^
60s (n = 104)	30.86 ± 4.71	28.15 ± 5.42	28.98 ± 5.20	2.71 (95% CI, 2.39–3.04)	<0.001 ^b^
Sum	28.83 ± 5.15	26.54 ± 5.53	27.69 ± 5.34	2.29 (95% CI, 2.18–2.39)	<0.001 ^b^
Back area					<0.001 ^a^
20s (n = 183)	28.46 ± 5.05	27.54 ± 5.83	28.03 ± 5.42	0.92 (95% CI, 0.65–1.18)	<0.001 ^b^
30s (n =213)	27.96 ± 5.57	24.46 ± 5.49	26.23 ± 5.53	3.50 (95% CI, 3.27–3.74)	<0.001 ^b^
40s (n = 228)	28.96 ± 5.27	26.65 ± 5.85	27.75 ± 5.57	2.31 (95% CI, 2.05–2.56)	<0.001 ^b^
50s (n = 177)	29.97 ± 4.88	27.30 ± 5.14	28.28 ± 5.04	2.67 (95% CI, 2.43–2.91)	<0.001 ^b^
60s (n = 104)	31.06 ± 4.62	28.09 ± 5.44	29.00 ± 5.19	2.97 (95% CI, 2.67–3.26)	<0.001 ^b^
Sum	28.90 ± 5.18	26.50 ± 5.61	27.70 ± 5.38	2.40 (95% CI, 2.34–2.46)	
Lateral-side area					<0.001 ^a^
20s (n = 183)	28.09 ± 5.06	27.05 ± 5.73	27.60 ± 5.37	1.04 (95% CI, 0.87–1.20)	<0.001 ^b^
30s (n =213)	27.25 ± 5.68	24.45 ± 5.40	25.89 ± 5.54	2.79 (95% CI, 2.61–2.98)	<0.001 ^b^
40s (n = 228)	27.84 ± 5.56	26.52 ± 5.77	27.15 ± 5.67	1.32 (95% CI, 1.07–1.57)	<0.001 ^b^
50s (n = 177)	28.67 ± 5.43	27.16 ± 5.19	27.71 ± 5.28	1.51 (95% CI, 1.23–1.78)	<0.001 ^b^
60s (n = 104)	29.34 ± 5.54	27.69 ± 5.54	28.21 ± 5.54	1.65 (95% CI, 1.29–2.00)	<0.001 ^b^
Sum	27.99 ± 5.45	26.29 ± 5.55	27.18 ±5.49	1.70 (95% CI, 1.53–1.87)	
Sole area					<0.001 ^a^
20s (n = 183)	25.97 ± 4.35	25.46 ± 4.76	25.73 ± 4.54	0.52 (95% CI, 0.09–0.94)	0.019 ^b^
30s (n =213)	26.46 ± 5.13	23.30 ± 4.80	24.90 ± 4.97	3.16 (95% CI, 2.79–3.53)	<0.001 ^b^
40s (n = 228)	26.47 ± 4.96	24.91 ± 5.37	25.65 ± 5.17	1.57 (95% CI, 1.14–2.00)	<0.001 ^b^
50s (n = 177)	27.47 ± 4.93	25.75 ± 5.12	26.38 ± 5.02	1.72 (95% CI, 1.35–2.10)	<0.001 ^b^
60s (n = 104)	27.49 ± 5.19	26.22 ± 5.26	26.61 ± 5.24	1.26 (95% CI, 0.76–1.77)	<0.001 ^b^
Sum	26.59 ± 4.87	24.85 ± 5.03	25.74 ± 4.98	1.74 (95% CI, 1.70–1.77)	
All areas					<0.001 ^a^
20s (n = 183)	27.75 ± 4.87	26.89 ± 5.50	27.34 ± 5.17	0.86 (95% CI, 0.59–1.12)	<0.001 ^b^
30s (n =213)	27.41 ± 5.47	24.19 ± 5.26	25.82 ± 5.37	3.22 (95% CI, 2.82–3.62)	<0.001 ^b^
40s (n = 228)	28.03 ± 5.24	26.21 ± 5.71	27.07 ± 5.48	1.82 (95% CI, 1.77–1.87)	<0.001 ^b^
50s (n = 177)	28.98 ± 5.04	26.88 ± 5.15	27.65 ± 5.11	2.10 (95% CI, 1.83–2.37)	<0.001 ^b^
60s (n = 104)	29.70 ± 5.02	27.54 ± 5.42	28.20 ± 5.29	2.16 (95% CI, 1.88–2.44)	<0.001 ^b^
Sum	28.08 ± 5.16	26.05 ± 5.43	26.97 ± 5.31	2.03 (95% CI, 1.74–2.32)	<0.001 ^b^

CI: confidence interval. ^a^ ANOVA between five age groups; ^b^ paired *t*-test between both sexes.

## Data Availability

The data presented in this study are available on request from the corresponding author. The data are not publicly available due to the privacy of participants.
